# Do the political attitudes of students change during their time in higher education?

**DOI:** 10.1007/s10734-022-00915-8

**Published:** 2022-09-13

**Authors:** Tom Fryer

**Affiliations:** Manchester Institute of Education, University of Manchester, Manchester, UK

**Keywords:** Higher education, Political attitudes, Graduate outcomes, Politics, Student politics

## Abstract

Experience of higher education (HE) has come to characterise many contemporary political divisions, including those related to Brexit, Trump and coronavirus policy. However, the academic literature is unclear whether HE plays a causal role in changing peoples’ political attitudes or is simply a proxy. Furthermore, in many contexts, there is limited descriptive evidence on whether students’ political attitudes change during HE. This paper focuses on the UK, using data from the British Election Study, to make a twofold contribution. Firstly, the paper introduces recent political science theorising on the nature of contemporary political divisions, which has remained largely outside the HE literature to date. This theorising is illustrated through a cross-sectional analysis, comparing the political attitudes of those with and without experience of HE, showing that the former tend to be more left-leaning and less ethnocentric. Secondly, a longitudinal analysis is performed to assess how students’ political attitudes change during their time in HE. While in HE, students tend to make small movements to the left and become less ethnocentric, representing approximately 20–33% of the overall division between those with and without experience of HE. These findings are interpreted through a critical realist lens—they evidence that HE *could* have a causal role to play in creating contemporary political divisions. However, to establish whether HE does play a causal role, further intensive research is needed to explore how particular aspects of HE might bring about these changes and how this varies for different students in different contexts.

## Introduction

Experience of higher education (HE) has come to characterise many contemporary political divisions, including those related to Brexit, Trump and coronavirus policy. Broadly speaking, we tend to find younger, urban-dwelling graduates on the one hand, and older, town-dwelling non-graduates on the other (Sobolewska & Ford, 2020). Similarly, universities are increasingly talked about in relation to the so-called culture wars, with claims that professors force polarising political attitudes onto students which contributes to contemporary political divisions. Thus, experience of higher education (HE) has become an important topic within recent political science research and theorising. However, little of this research has made its way into HE-specific literature (Yang & Hoskins, 2020). This is an important gap, preventing the HE literature from engaging with wider political science research, and also hindering political science from understanding how HE might play a causal role in contributing to these divisions.

Of this political science literature, a useful historical and macro-level analysis is given by Piketty (2018) who compares how political conflict in Britain, France and the USA has evolved from 1948 to 2017. He concludes that there has been a gradual shift from political divisions characterised by class-based interests to those based on identity. Divisions that were previously characterised by low-income voters supporting left-leaning parties and high-income voters supporting right-leaning parties have shifted to be more identity-based, as voters with higher levels of education support the left and those with less education support the right. This shift can be seen in the UK, as in the 1950s, graduates were approximately 25 percentage points less likely to vote for the left-leaning Labour Party compared with non-graduates, whereas from 2015, graduates were more likely to vote for Labour than non-graduates (Piketty, 2018).

Beyond these macro-level analyses, other political science work has looked in more detail at the nature of contemporary political divisions. For example, Sobolewska and Ford’s (2020) *Brexitland* argues that the contemporary divides in the British electorate have been influenced by two long-term trends: first, the rising number of graduates, and second, the rising number of people from ethnic minority groups. In the 1960s, only about 10% of people attended HE and the ethnic minority population was less than 1%, whereas this has increased to over 40% of people attending HE and an ethnic minority population of over 12% in the 2010s. Sobolewska and Ford (2020) discuss how contemporary electoral divides are increasingly based on voters’ ethnocentric identities, with primarily low-education *identity conservatives* on the one hand, and high-education *identity liberals* and ethnic minority *necessity liberals* on the other. In other words, the UK’s political divides have become less concerned with the economy and public service delivery, and more concerned with topics related to ethnocentrism, such as immigration. Both the Brexit referendum and the 2019 election were characterised by these divides in voters’ ethnocentrism, as those with more ethnocentric worldviews tended to favour Leave and the Conservative Party, while those with less ethnocentric views favoured Remain and the Labour Party.

Although HE has featured prominently within recent political science research and theorising, this has not been accompanied by an increased focus within the HE literature. Within the HE literature, the impact of HE on students’ politics remains an under-developed area of study (Raaper, 2020). There is a large HE literature that considers the role of HE in citizenship (Yang & Hoskins, 2020), and a growing number of studies on capabilities (Walker, 2008), but it is rare for research to directly tackle HE’s impact on political attitudes—perhaps this hesitancy stems from a belief that HE should promote outcomes that are seen as ‘objectively’ good, whereas political attitudes are often perceived as ‘subjective’ beliefs.

Of the HE literature that does directly address the topic of HE and students’ politics, the majority is focussed on the USA. Several studies have documented the views of students and graduates, who are found to be more liberal than their peers who did not go to HE (Campbell & Horowitz, 2016; Castro Samayoa et al., 2018; Pascarella et al., 2012). However, there are still significant gaps in establishing the role of HE (Strother et al., 2020). Indeed, the literature is yet to move away from a central question of whether HE is causal or simply a proxy (Dodson, 2014), a debate that has been around from at least the 1950s (Nogee & Levin, 1958). This academic attention in the USA has led to a series of national surveys of students during their time in HE by the Higher Education Research Institution (HERI) at the University of California Los Angeles (Higher Education Research Institute, 2022). This is a valuable data source that is used by many studies to look at the impact of HE on students’ politics (Castro Samayoa et al., 2018; Dodson, 2014). Outside of the USA, the literature is less developed (Strother et al., 2020; Yang & Hoskins, 2020) and even the descriptive patterns in graduates’ politics are less well documented (Sloam et al., 2021). One exception to this is China, in which several studies have been attentive to the ways in which campus culture and graduate political engagement have shifted after 1989 (Chan, 1999; Liu & Shen, 2021; Xu, 2004).

This paper adopts a critical realist approach, as is explained in the next section, to assess the current evidence for whether HE has a causal role in the political divisions described by political science research. From this critical realist perspective, our causal knowledge would consist of an explanation of *how* HE led to a change of political attitudes, for example through a particular course or extra-curricular activity. This type of causal evidence is currently limited, as there are a lack of in-depth studies that explore how HE impacts political attitudes (Castro Samayoa et al., 2018). Hence, despite the prominence that Sobolewska and Ford, (2020) give to HE in their explanations of contemporary political divides in the UK, their book features no reference to a study that demonstrates how HE creates these political divisions. This is particularly notable as the authors explicitly argue that the rising participation in HE is one of the two key causal mechanisms that explain the new political divisions. Much political science literature takes it as a given that HE has a causal role to play, but as of yet, there is little HE-specific research to support or deny this assumption.

This paper aims to help address this gap by assessing how students’ political attitudes change during their time in UK HE using data from the British Election Study (BES). The UK is used as a case study to address the limited number of non-US analyses. This paper is aware of no other study that has used the BES to address primarily educational questions, and it is hoped that this helps introduce the dataset to a new field of research. The paper begins with a cross-sectional analysis of the political attitudes of those with and without HE, illustrating the recent political science theorising on political divisions. The paper then uses a longitudinal analysis examining how students’ political attitudes and identities change during their time in HE. This is an important first step in the process of assessing whether HE does have a causal impact—if students do not change their political attitudes during HE, it is unlikely that HE has a causal role in contemporary divisions.

## Methodology

### Theoretical framework

This paper is informed by critical realism (Archer, 2003; Bhaskar, 2008). This philosophy asserts that causation is not concerned with constant conjunctions of events in which causes have constant quantifiable impacts. Instead, critical realism asserts that causes are the powers of things, and when multiple powers operate at the same time, causes only *tend* to bring about events. This position is held in opposition to positivism’s search for universal laws. Although little contemporary research would claim to seek universal laws, research that presents the output of regression modelling and asserts this constitutes an explanation is informed by positivistic assumptions (Porpora, 2015). Critical realism explains that the end-point of our analysis should be nuanced causal explanations—in the words of Pawson and Tilley (1997), we should seek to explain *how* causes work, who they work for and in what circumstances. Importantly, as Margaret Archer (2003) stresses, these explanations must focus on how reflexive agents act and respond to their objective situations. The output of a regression model can do none of this. For the topic of HE’s impact on students’ politics, this means that we should ultimately seek nuanced explanations of how (if at all) particular aspects of HE tend to cause certain changes in students’ politics, and how this plays out for different students, with different projects, in different contexts.

Critical realism can also help identify which methodologies are appropriate in different research contexts. Danermark et al. (2001) distinguish between *extensive* and *intensive* research approaches—the former provide broad descriptions of a topic and are useful in under-developed research areas, whereas the latter produce nuanced causal explanations that are more appropriate in better-researched contexts. In our case, the impact of HE on students’ politics is an under-developed area of study (Yang & Hoskins, 2020), making extensive research approaches the most appropriate. In recognition of this, I firstly conducted a descriptive cross-sectional analysis. The aim was to illustrate the divisions between those with and without HE for some of the most important political attitudes, but the question of what causes these divisions cannot be answered by this extensive research. The subsequent longitudinal analysis takes an initial step towards assessing the causal role of HE, by looking at how students’ political attitudes change during their time in HE. While this does not provide a nuanced causal explanation of *how* HE impacts students’ politics, it represents a first step in assessing whether HE plays a causal role or not; if students’ political attitudes do not change during their time in HE, this implies that HE is not causal.

### Data

This paper uses the BES panel survey data (Fieldhouse et al., 2020). As a panel survey, the BES aims to survey the same people over time, and the survey is conducted online by YouGov with approximately 30,000 people, with weightings applied to make the sample representative of the UK population. It has been in operation since 2014 and currently has 20 waves. Although this survey does not primarily aim to assess the links between HE and political attitudes, it does collect a wide range of data on political attitudes as well as information on educational level. All analysis is completed in R, using the svy package (Lumley, 2021).

### Cross-sectional analysis

I conducted a cross-sectional analysis, comparing the political attitudes and identities of those with and without experience of HE using Wave 20 of the BES survey, administered between 3 June 2020 and 21 June 2020. This was the latest available data at the point of analysis. The cross-sectional analysis involved the following variables:

1. Experience of HE. The BES survey asked if people had ever attended a university or HE institution, with five possible answers: No, I have never attended HE; Yes, I am currently enrolled in HE; Yes, but I didn’t complete HE; Yes, I graduated from HE; and Don’t know. I constructed a binary variable: (1) those with experience of HE (i.e. current students and graduates) and (2) those without experience of HE (i.e. those who said they had never attended HE). Those who answered ‘Don’t Know’ or ‘Yes, but I didn’t complete HE’ were excluded, the former because it is unclear how to interpret, and the latter as we do not know how long they spent in HE. This excluded 3251 responses out of 31,468 (10.3%).
2. Age. Respondents report their age, and this was converted to the following categories: Under 25; 26–35; 36–45; 46–55; 56–65; and 66 + .
3. Left–right identity. Respondents report their political identity on an 11-point scale, where 0 was labelled ‘Left’ and 10 was labelled ‘Right’, or answered ‘Don’t know’. From this scale, four categories were created: 0 to 3 were labelled ‘Left’; 4 to 6 were labelled ‘Centre’; 7 to 10 were labelled ‘Right’; and ‘Don’t know’ was kept the same.
4. Ethnocentrism. Respondents report their attitude to whether ‘immigration undermines or enriches Britain’s cultural life’ on an 7-point scale, where 1 is the most anti-immigration position and 7 the most pro-immigration. Respondents could also answer ‘Don’t know’. From this scale, four categories were created: 1 to 3 were labelled ‘Anti-immigration’; 4 was labelled ‘Centre’; 5 to 7 were labelled ‘Pro-immigration’; and ‘Don’t know’ was kept the same.^1^

The first variable captures people’s experience of HE, as this is the main focus of the paper. Age is included in the cross-sectional analysis, as access to HE has varied between generations and age is a key variable in contemporary political divisions (Sobolewska & Ford, 2020). The two outcome variables are chosen in light of recent political science theorising on the relative importance of class-based and identity-based divides. The identity on a left–right scale aims to capture attitudes to the economy and public service delivery, reflecting more traditional class-based politics (Piketty, 2018). The attitude to immigration is used to as proxy for the ethnocentrism of respondents, following the approach of Sobolewska and Ford (2020).^2^

The cross-sectional analysis used these two independent variables (experience of HE and age) to calculate the percentage of each group that held a particular political attitude. For each political attitude, the percentage point difference between those with and without experience of HE was then calculated for each age group. The mean of these differences for each political attitude was then calculated. A chi-squared test was performed on these means, to indicate whether these differences were due to chance. Furthermore, an overall mean difference between those with and without experience of HE, as stratified by age, was generated by treating the outcome variables as numerical rather than categorical data.

### Longitudinal analysis

I also conducted a longitudinal analysis of how students’ political attitudes changed during their time in undergraduate HE programmes, enabled as the BES is a panel survey of the same people over time. This analysis required the creation of a subpopulation of students, as the BES includes many non-students. Given that the specific variable on HE attendance was only introduced into the BES in Wave 7, this paper used data from 14 waves of the BES (Wave 7 to Wave 20), which were conducted from April 2016 to June 2020. The following inclusion criteria were used: individuals must have answered the panel survey over three consecutive academic years and reported themselves to be in HE throughout this time. This means that the subpopulation captured four cohorts of students: the first that attended HE from 2015 to 2018, the second from 2016 to 2019, the third from 2017 to 2020 and the fourth from 2018 to 2021 (see Table 3, Appendix). This requirement of three consecutive years allows a judgement of how students’ political attitudes changed over time; this time interval was chosen because it corresponds with the conventional length of a full-time undergraduate programme in much of the UK (see the “Limitations” section, for a further discussion).

To create this subpopulation, the first step was to apply the inclusion criteria. This was achieved by creating a number of different cohorts, e.g. limiting the data to those who had answered the survey in each academic year from 2015 to 2018, and reported themselves to be in HE throughout this time, to create the first cohort (see Table 3, Appendix). If a student answered multiple BES surveys in a single academic year, the first survey response was taken to represent their position. Within each cohort, the variables for left–right identity were re-named to reflect the academic year of study that it represented, for example in the first cohort, ‘leftRightWave7’ was re-named ‘leftRightYear1’; ‘leftRightWave10’ became ‘leftRightYear2’; and ‘leftRightWave14’ became ‘leftRightYear3’. With these shared variable names, the different cohorts could be recombined, creating a subpopulation of students who had one point of data for each of their three years in HE, i.e. variables representing left–right identity in Year 1, Year 2 and Year 3, and the equivalent for ethnocentrism. This subpopulation was deduplicated to avoid students on four-year courses appearing more than once within the analysis, e.g. if a student began a four-year course in 2015, they would appear twice as they were in HE for both 2015–2018 and 2016–2019—the latter student data was deleted. A total of 574 students were included in this combined subpopulation, although due to missing data, only 531 out of 574 students had data on ethnocentrism. BES weights were not applied to the data for the longitudinal analysis as it did not aim to be nationally representative. The longitudinal analysis involved the following variables:

1. Left–right identity in Year 1, Year 2 and Year 3. Respondents report their political identity on an 11-point scale, where 0 was labelled ‘Left’ and 10 was labelled ‘Right’, or reported ‘Don’t know’, in each of their three years in HE. The same categories were created as in the cross-sectional analysis.
2. Difference in left–right identity between Year 1 and Year 3. This variable was calculated by excluding those who answered ‘Don’t know’ in Year 1 or Year 3, and then subtracting the reported identity in Year 1 from the identity in Year 3. For example, if a student reported an identity of 4 in Year 1 and 3 in Year 3, then the difference was − 1, representing a 1-point move to the left.
3. Ethnocentrism in Year 1, Year 2 and Year 3. Respondents report their attitude to whether ‘immigration undermines or enriches Britain’s cultural life’ on an 7-point scale, where 1 is the most anti-immigration position and 7 the most pro-immigration, or reported ‘Don’t know’, in each of their three years in HE. The same categories were created as in the cross-sectional analysis.
4. Difference between Year 1 and Year 3 on ethnocentrism. This variable was calculated by excluding those who answered ‘Don’t know’ in Year 1 or Year 3, and then subtracting the reported identity in Year 1 from the identity in Year 3.

The longitudinal analysis considered the same two political attitude outcomes as the cross-sectional analysis: left–right identity and ethnocentrism. The analysis began by considering how the subpopulation changed their political attitudes over the course of their three years in HE. Descriptive statistics for the percentages of students reporting different political attitudes in each of their three years and percentage point differences between Year 1 and Year 3 were calculated. Furthermore, a mean position on the scale was also calculated for each of the three years, and the change between Year 1 and Year 3 was calculated—this treated the outcome variables as numerical data. The analysis subsequently went on to assess the extent of individual level change in political attitude, not merely average changes in the subpopulation over time. By subtracting the position on the scale in Year 1 from the position in Year 3, a calculation of individual change can be made. This individual level analysis complements the average analysis, as it is possible there is lots of individual variation, but this largely cancels out. Polynomial contrast tests were used to assess the significance of trends in individuals’ political attitudes over time—this involved a generalised linear model with the following variables: outcome (left–right identity or ethnocentrism), an ID variable and a time variable that captured the year of HE.

### Limitations

There are limitations to this analysis, many of which stem from the fact the BES does not primarily aim to answer questions that link education and political attitudes. In the UK, there is no equivalent to the HERI data from the USA. This means a limited amount of education-related data is collected, for example information on the type of HE institution attended or the subject studied is either not available or inconsistently captured. As students’ experiences in different institutions and subject areas can vary a great deal, this could lead to different impacts on students’ political attitudes. Another limitation is that the two outcome variables (left–right and ethnocentrism) are related—your ethnocentric beliefs are likely to influence your identity on a left–right scale, with left-leaning positions associated with less ethnocentric perspectives and right-leaning with more ethnocentrism. Treating left–right identities as indicative of economic attitudes, and ethnocentric identities as indicative of cultural attitudes, is a simplification.

The longitudinal analysis has other limitations. Firstly, the BES does not always capture data at the start of an academic year and surveys were administered at irregular time intervals. For example, students that began HE in September 2017 were first interviewed in May 2018, meaning that most would have largely completed their first year of courses. If students do undergo changes in political attitudes, and especially if this occurs early during their time in HE, then this analysis would underestimate the change. Secondly, the subpopulation was created by looking for survey respondents with three consecutive years in HE, which corresponds to the most common length of a full-time undergraduate degree in the UK. However, this approach could lead to an underestimation of the changes in political opinion that occur during HE, as some students will be on courses that last longer than three years, whether they are part-time or on a longer degree programme. Furthermore, although the subpopulation is likely to mostly consist of undergraduate students, it may also include those on postgraduate teaching and research degrees—for example, if a student begins answering the BES in their second year of a three-year undergraduate programme, and then continues immediately to a postgraduate programme upon graduating, then this student’s three years would correspond to (1) year 2 of undergraduate, (2) year 3 of undergraduate and (3) year 1 of postgraduate. Given this limitation, this paper discusses ‘students’ and their time in HE, rather than specifically ‘undergraduates’ or ‘postgraduates’.

## Results and discussion

### Cross-sectional analysis: political divisions by experience of HE

**Fig. 1:**
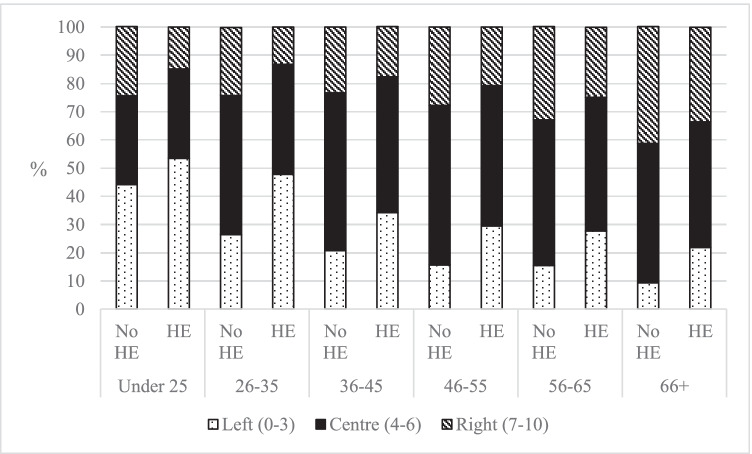
Self-identity on a left–right scale, by experience of HE and age (author’s calculation, BES Wave 20)

This cross-sectional analysis explores the differences in political attitudes and identities between those with and without experience of HE, reflecting the increasing importance of this division in contemporary politics. The first aspect of political identity considered is left–right identity. Figure 1 shows the spread of these left–right identities, excluding those who answered ‘Don’t Know’ to aid the comparisons within age groups. Including those who answered ‘Don’t Know’ does not change the macro-patterns from Fig. 1 (see Table 4, Appendix).

Figure 1 shows that those with experience of HE were more likely to identify on the left, compared with those with no experience of HE. The average difference between those with and without HE for different age groups identifying on the left was 13.8 percentage points. A smaller difference was seen for those identifying on the right—those with experience of HE were on average 8.2 percentage points less likely to identify on the right, compared with those without experience of HE in the same age group. Those with HE were less likely to respond ‘Don’t Know’—the average gap between the different age groups was 17.8 percentage points. This could be explained as those with experience of HE tend to have higher rates of reported knowledge, attention and self-efficacy, which has been documented elsewhere in the literature (Holland, 2009). The differences between those with and without HE for each age group were all significant (Under 25 *p* < 0.05, all other ages *p* < 0.001, see Table 5, Appendix). This statistically significant result should not be interpreted to mean that experience of HE causes these differences; it is perfectly possible that a significant difference is caused by something unrelated to HE. Overall, the finding that those with experience of HE were more likely to be left-leaning is in-keeping with the existing literature (Campbell & Horowitz, 2016; Castro Samayoa et al., 2018; Pascarella et al., 2012).

However, it is important not to overstate these patterns. Those with experience of HE were more likely to be left-leaning and less likely to be right-leaning, only when compared to those without experience of HE *from the same age group*. This is different to the claim that those with HE were more left- than right-leaning, which is unsupported by the data. If we look at the 66 + age group, only 21.9% identified as left-leaning whereas 33.5% identified as right-leaning; it is only, *compared to 66* + *year olds without HE*, that graduates in the 66 + age group were more left-leaning and less right-leaning. Similarly, Fig. 1 shows that it was common for people to identify with a position in the centre of the scale—the centre was the most popular position for all groups, with the exception of Under 25s with HE, Under 25s without HE and 26–35s with HE, all of whom favoured left-leaning positions. Therefore, apart from the younger generations that skewed more left-leaning, other age groups tended to identify with more centrist positions regardless of whether they had experienced HE or not.

**Fig. 2:**
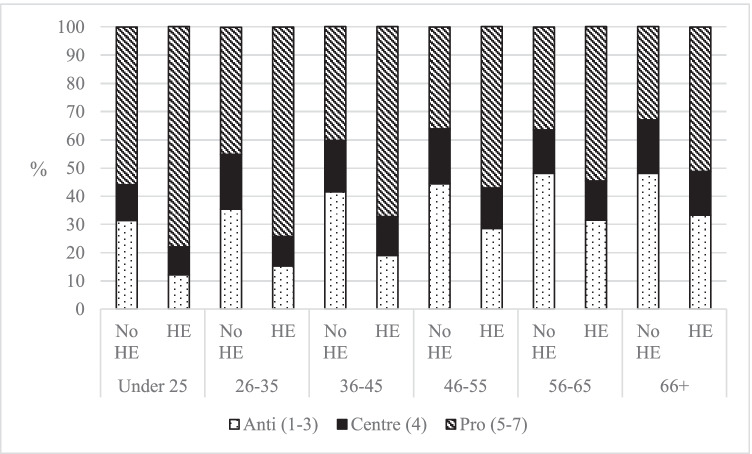
Attitudes to the cultural impact of immigration, by experience of HE and age group (author’s calculation, BES Wave 20)

The second political attitude considered in this analysis relates to ethnocentrism. Specifically, attitudes on the cultural impacts of immigration were analysed, which is a widely used proxy for ethnocentrism (Sobolewska & Ford, 2020). The findings are summarised in Fig. 2. Again, to aid legibility, the figure excludes those who answered ‘Don’t Know’, although this does not influence the trends (see Table 6, Appendix).

Figure 2 demonstrates that those with experience of HE were more likely to report positive attitudes to the cultural impacts of immigration, and less likely to report negative attitudes, when compared to those without experience of HE from the same age group. For example, in the 46–55 age group, 57.1% of those with HE held positive views, compared with 36.1% of those without HE. On average across all the age groups, those with experience of HE were 22.6 percentage points more pro-immigration and 18.2 percentage points less anti-immigration, compared with those without experience of HE from the same age group. The differences between those with and without HE for each age group were all significant (*p* < 0.001, see Table 7, Appendix) when chi-squared tests were performed.

Figure 2 does mask some important trends. Those with experience of HE were much more likely to report the highest possible pro-immigration attitudes (7 on the scale)—apart from those aged 66 + , the most common score for all age groups with experience of HE was 7, demonstrating the strongly held opinions. Similarly, those with experience of HE were much less likely to hold a very strong anti-immigration attitude (1 on the scale), compared with those without HE in the same age group—this was particularly pronounced in the under 25s (3.1% vs 11.5%) and 26–35s (4.5% vs 16%). This finding corroborates Sobolewska and Ford’s (2020) argument that ethnocentrism and the corresponding attitudes to immigration are often framed as a moral issue—by thinking in terms of moral rights and wrongs, people tend to be pushed to towards the ends of the scale. Specifically, Sobolewska and Ford (2020) argue that identity liberals (those with HE) are more likely to see anti-immigration as morally wrong, whereas this is contested by identity conservatives.

Overall, this cross-sectional analysis has illustrated that those with experience of HE tend to hold more left-leaning identities and less ethnocentric attitudes, compared with those without experience of HE of the same age. The scale of the percentage point differences was similar for both the left–right and ethnocentric scale—those with experience of HE were on average 13.8 percentage points more left-leaning and 22.6 percentage points more pro-immigration, compared with to those without experience of HE from their age group. This illustrates the way that experience of HE has become a key characteristic of our contemporary political divisions. However, there were some differences between the patterns in left–right identity and ethnocentrism. While left–right identities were concentrated around the centre, the attitudes to immigration were more polarised, with many people, especially those with experience of HE, strongly believing that the cultural impacts of immigration were positive for the UK.

### Longitudinal analysis: changes in students’ politics during their time in HE

**Fig. 3:**
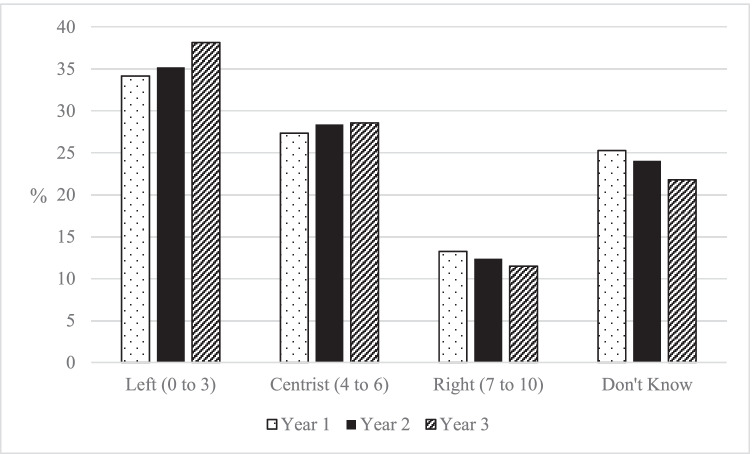
Self-identity of students on a left–right scale, by year of study in HE (author’s calculation, BES Waves 7–20)

The longitudinal analysis assessed whether students’ political attitudes change during their time in HE, taking a first step to assess whether HE has a causal role to play in creating contemporary political divisions. The changes in left–right identity of students over their three years in HE are shown in Fig. 3. There appeared to be a small increase in the percentage of students that identified on the left, with 34.1%, 35.2% and 38.2% in Years 1, 2 and 3, respectively. This seemed to correspond to a drop in the percentage of students reporting ‘Don’t Know’, at 25.3%, 24.0% and 21.8%, and a smaller drop in the percentage of students reporting ‘Right-leaning’ 13.2%, 12.4% and 11.5%, respectively.

However, amongst these changes, Fig. 3 shows a reasonable amount of stability. While there was a tendency to move to the left, the percentage of students holding left, right and centrist positions remained relatively stable. Furthermore, by comparing the mean change in the 11-point left–right scale from Year 1 to Year 3, there was only a 0.2-point movement to the left.

**Fig. 4:**
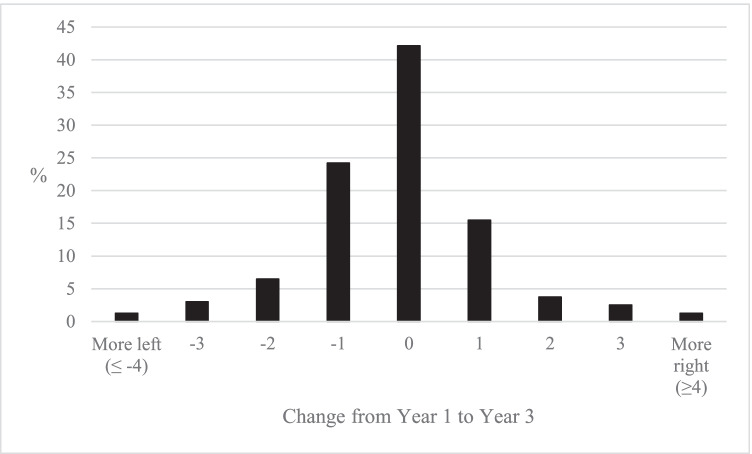
Percentage of students that change self-identity on left–right scale from year 1 to year 3 (author’s calculation, BES Waves 7–20)

It is possible that there are large individual changes in left–right identity during HE as students shift from right to left and left to right, but that these individual changes largely cancel themselves out in the average changes considered in Fig. 3. To explore change at an individual level, Fig. 4 compares the reported positions of the same student in Year 1 and Year 3.^3^ This analysis shows that it was common for individual students not to change their left–right identity from Year 1 to Year 3 (42.1%). This demonstrates that there was little change for many students, a fact further illustrated by the fact that 81.8% of students moved one point or less on the left–right scale during their three years in HE. It was rare for students to radically change their political stance, with only 1.6% of all students moving from a position on the right in Year 1 to one on the left in Year 3, and 1.4% making the opposite move from left to right. Amongst this stability, the individual level analysis also shows a slight tendency for students to move in a left-leaning direction during HE: while 34.9% of students moved in a left-leaning direction, 22.9% moved in a right-leaning direction. A polynomial contrast test for the significance of these trends resulted in a significant result for a linear pattern (*p* < 0.05, see Table 8, Appendix).

**Fig. 5:**
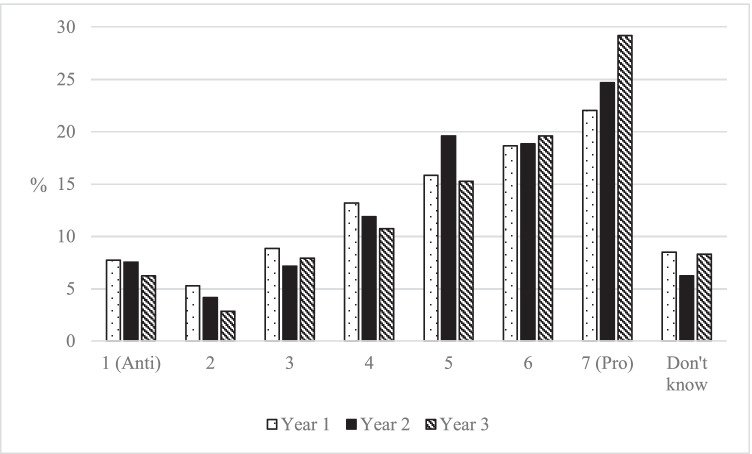
Students’ attitudes to the cultural impact of immigration, by year of study in HE (author’s calculation, BES Waves 7–20)

The second political attitude considered in the longitudinal analysis was attitude to the cultural impacts of immigration. Figure 5 demonstrates that there was a tendency for students to move to more pro-immigration and less ethnocentric positions during their time in HE. Compared with Year 1, students in Year 3 were more likely to give the most positive answer about the cultural impacts of immigration (a score of 7), which increased from 22.0 to 29.2%. There were corresponding falls in the number of students who gave answers that reflect more anti-immigration attitudes from Year 1 to Year 3. Those giving a score of 1 or 2 dropped from 7.7 to 6.2% and from 5.3 to 2.8%, respectively. If we compare the changes in the mean from Year 1 to Year 3, there was an average 0.4-point movement to more pro-immigration positions (on an 7-point scale).

**Fig. 6:**
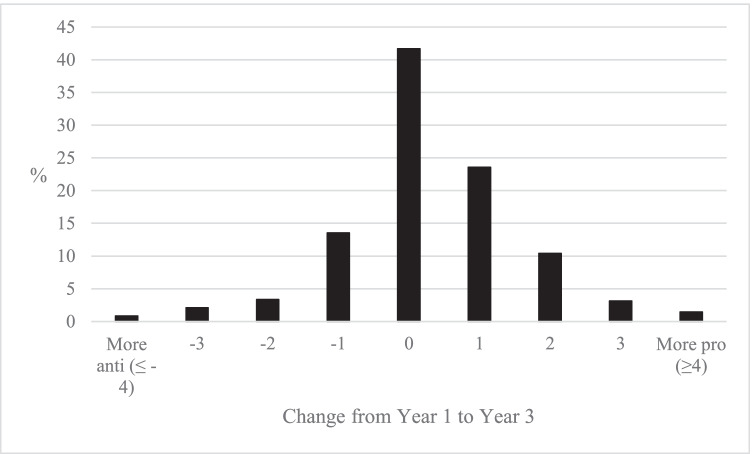
Percentage of students that change attitudes to the cultural impacts of immigration from year 1 to year 3 (author’s calculation, BES Waves 7–20)

Figure 6 considers the changes in individual students’ ethnocentrism from Year 1 to Year 3, as the extent of individual change may be underestimated in Fig. 5. As with the left–right changes, the overall pattern was one of relative stability in students’ attitudes. Between Year 1 and Year 3, 78.7% of students changed by one-point or less on the 7-point scale, and it was most common for students not to change their position (41.7%). Similarly, rarely did a student move from anti- to pro-immigration (4.1% of all students) or pro- to anti-immigration (2.1% of all students). Amongst this stability, there was a tendency for students to move to more pro-immigration positions: 38.5% of students moved to more pro-immigration positions, while 19.7% moved to be more anti-immigration positions. A polynomial contrast test for the significance of these trends resulted in a significant result for a linear pattern (*p* < 0.001, see Table 9, Appendix).

How do the changes in students’ ethnocentrism compare with their changes in left–right identity? The average changes in the left–right identity were smaller than the changes in ethnocentrism. If we compare the changes in the mean from Year 1 to Year 3, there was a 0.2-point movement to the left (11-point scale) and a 0.4 movement to pro-immigration (7-point scale). The larger comparative changes in ethnocentrism are also seen in the percentages of students identifying with different positions. While there was a 3.1 percentage point increase in students identifying as left-leaning and a 3.0 percentage point decrease in students identifying as right-leaning from Year 1 to Year 3, the corresponding changes for ethnocentrism were an 8.1 percentage point increase in students reporting positive attitudes to immigration and a 5.4 percentage point decrease in those reporting negative attitudes.

### Comparing the size of the changes in students during HE, with the overall political divisions

The remainder of this section will discuss the cross-sectional and longitudinal analyses, in order to compare the size of the political divisions found in the cross-sectional analysis, with the size of the changes students make during HE in the longitudinal analysis. This comparison will help put the size of change in students’ political identities during their time in HE into context.

Compared with the overall division between those with and without experience of HE, the changes in students over their time in HE were small but significant. In terms of left–right identity, whereas the average gap between those with and without HE is 1.0 points to the left, the mean identity of students moved 0.2 points left from Year 1 to Year 3 (see Table 1). In other words, the mean movement in left–right identity during HE represents 20% of the overall gap between those with and without HE (for more detail see Table 10, Appendix).

**Table 1:** Changes in left–right identity of students from Year 1 to Year 3, compared with the average difference between those with and without experience of HE for different age groups

Changes in left–right identity of students from Year 1 to Year 3		Average difference in left–right identity between those with HE and without HE for different age groups		Student change as a % of the overall difference
Mean movement (scale from 0 = left to 10 = right)	− 0.2	Mean difference (scale from 0 = left to 10 = right)	− 1.0	20.0
Changes in those identifying as ‘Left’ (% points)	+ 3.1	Average difference in those identifying as ‘Left’ (% points)	+ 13.8	22.5
Changes in those identifying as ‘Centre’ (% points)	− 0.1	Average difference in those identifying as ‘Centre’ (% points)	− 5.6	1.8
Changes in those identifying as ‘Right’ (% points)	− 3.0	Average difference in those identifying as ‘Right’ (% points)	− 8.2	36.6

This comparison between the overall gaps between those with and without experience of HE and the change of students during their time in HE can also be made by looking at the percentage of people that identify with different positions. Those with HE were on average 13.8 percentage points more likely to identify on the left, and 8.2 percentage points less likely to identify on the right, compared with those without HE in the same age group (see Table 1 and Table 11, Appendix). These percentage point differences of + 13.8 and − 8.2 were larger than the differences in students over their time in HE, which were + 3.1 and − 3.0. To put this into context, 3.1 percentage points represent 22.5% of the 13.8 percentage point gap between those with and without HE experience.

In terms of ethnocentrism, there was an average difference of 1.2 points between those with and without HE for different age groups, and there was a 0.4-point movement to be less ethnocentric in the mean score of students from Year 1 to Year 3 (see Table 2). In terms of different attitudes, those with experience of HE were on average 22.6 percentage points more pro-immigration and 18.2 percentage points less anti-immigration, compared with those without experience of HE from the same age group (see Table 2 and Table 12, Appendix). Again, this was larger than the differences that are observed in students over their time in HE, at + 8.1 and − 5.4 percentage points, respectively. To put this into context, 8.1 percentage points represent 35.9% of the 22.6 percentage point gap between those with and without HE experience.

**Table 2:** Changes in attitudes to cultural impact of immigration of students from Year 1 to Year 3, compared with the average difference between those with and without experience of HE for different age groups

Changes in ethnocentrism of students from Year 1 to Year 3		Average difference in ethnocentrism between those with HE and without HE for different age groups		Student change as a % of the overall difference
Mean movement (scale from 1 = anti to 7 = pro-immigration)	+ 0.4	Mean difference (scale from 1 = anti to 7 = pro-immigration)	+ 1.2	33.3
Changes in those identifying as ‘Pro-immigration’ (% points)	+ 8.1	Average difference in those identifying as ‘Pro-immigration’ (% points)	+ 22.6	35.9
Changes in those identifying as ‘Centre’ (% points)	− 2.7	Average difference in those identifying as ‘Centre’ (% points)	− 4.4	61.4
Changes in those identifying as ‘Anti-immigration’ (% points)	− 5.4	Average difference in those identifying as ‘Anti-immigration’ (% points)	− 18.2	29.7

These comparisons illustrate that although students do become more left-leaning and less ethnocentric during their time in HE, this represents only part of the variation between those with and without experience of HE. Roughly speaking, the changes to political attitudes during HE represent approximately a fifth to a third of the overall gap between those with and without HE. This provides some initial support for the idea that HE could have a causal role to play in the creation of contemporary political divisions. By finding that students do change attitudes during their time in HE, this retains the possibility that HE has a causal role—an absence of change would have challenged the idea HE was causal.

However, these comparisons also highlight that if HE is causal, it is likely to be one of a number of factors that influence the political divisions described in the cross-sectional analysis. The changes that take place during HE were smaller than the overall gaps between those with and without HE, implying that there must be other causes that contribute to the gap in political attitudes between those with and without HE. These other causal factors could influence people before HE, for example the impact of family or previous schooling. Similarly, these influences could occur after HE, perhaps the different jobs that graduates take up in the labour market. This also offers evidence against the idea that HE forces political attitudes onto students—although there is some movement in attitudes, this does not represent a majority of the gap between those with and without HE. Instead, this paper suggests that the influence of HE on political attitudes is likely to be at most one amongst many causes of contemporary political divisions.

Although the evidence base is limited, given the lack of in-depth and intensive studies (Castro Samayoa et al., 2018), it is worth reflecting on some of the potential ways *how* HE may play a causal role. Firstly, the educational role of HE may be important in changing political attitudes. There is initial evidence that has found associations between particular subject areas and political attitudes—for example, Fernandez (2021) found an association between political science and political participation. Secondly, the social context and influence of peers in HE may have an impact (Strother et al, 2020). Thirdly, particular extra-curricular activities may lead to changes in political attitudes—some studies have noted how engagement with Student Unions may shape students’ politics (Raaper, 2020). This is far from an exhaustive list, and is intended as a broad overview of some of the potential ways HE could impact political attitudes.

## Conclusion

HE is becoming increasingly central to descriptions of contemporary political divisions and features prominently in recent political science research and theorising. However, there has been less focus on this topic in the HE-specific literature—the impact of HE on political attitudes is an under-researched area (Yang & Hoskins, 2020). Of the HE-specific literature that does exist, it tends to be focussed on the USA and has yet to move away from a central question of whether HE is causal or simply a proxy (Dodson, 2014). This is an important gap, preventing HE literature from engaging with wider political science research, and also hindering political science from understanding the *how* HE might causally contribute to these divisions. There is a need for more HE-specific research that zooms in on these political divisions and unpacks the causal role of HE, if any.

The paper began by introducing recent political science theorising on contemporary political divisions to the HE literature, engaging with Piketty (2018) and Sobolewska and Ford (2020). The nature of these divisions was illustrated through a cross-sectional analysis of political attitudes, by experience of HE and age, using the latest BES data. It was shown that those with experience of HE were more likely to be left-leaning, compared with their peers without HE. Across all the age groups, those with experience of HE were on average 13.8 percentage points more likely to identify on the left, compared with those without HE. This broadly links to Piketty’s (2018) analysis that identified a tendency for high-education voters to support left-leaning parties. The second political attitude considered was attitudes to the cultural impacts of immigration, a proxy for ethnocentrism (Sobolewska & Ford, 2020). Again, a difference was found: those with experience of HE were 22.6 percentage points more pro-immigration than those without HE, as an average across different age groups.

After outlining the way HE characterises contemporary political divisions, the paper sought to speak back to the political science literature by taking a first step to explore the potential causal role of HE. The longitudinal analysis judged whether students’ political attitudes changed during their time in HE. Specifically, this analysis assessed whether students’ political attitudes to left–right identity and ethnocentrism changed during three years in HE. This was a first step in assessing the causal role of HE because if no changes were found, it would be unlikely that HE has a causal role.

Overall, it was found that there was a change in students’ political attitudes during their time in HE. In terms of left–right identity, there was a 3.1 percentage point increase in students identifying on the left, and a mean change of 0.2 points to the left on an 11-point scale between Year 1 and Year 3. In terms of ethnocentrism, there were larger changes, with an 8.1 percentage point increase in those identifying as pro-immigration, and a mean change of 0.4 points towards pro-immigration positions on an 7-point scale between Year 1 and Year 3. These changes represent between a fifth and a third of the overall divisions between those with and without experience of HE that were described in the cross-sectional analysis. These changes do not support the more extreme notions that professors force their political attitudes onto students—very few students move from right to left, or from anti to pro-immigration positions, with the majority varying by only one-point or less on a scale during their time in HE.

This evidence suggests that HE could play a causal role, as one cause amongst several, in creating the division in political attitudes between those with and without experience of HE. However, future intensive research is needed to assess whether HE does have a causal role, and if so *how* HE brings about these changes. This could build on existing research that has found associations between aspects of HE and political attitudes, but this research should adopt intensive approaches to assess how particular aspects of HE (e.g. certain subjects, pedagogies or extra-curricular activities) impact the political attitudes of students, and how this might vary for different attitudes, for different students, in different HE providers. Other research could consider whether *not* attending HE influences people’s political attitudes, i.e. does an identity of ‘non-graduate’ have an impact? There is also the normative question of whether HE should aim to impact students’ political attitudes, say by challenging political attitudes that stem from falsehoods. This future work would enable HE research to speak back to political science, giving nuanced explanations of how HE relates to contemporary political divisions.

## Appendix

**Table 3:** How the BES Waves were divided into cohorts to form the subpopulation

BES Wave	Date of Data Collection	Cohort 1 (2015−2018)	Cohort 2 (2016−2019)	Cohort 3 (2017−2020)	Cohort 4 (2018−2021)
7	Apr−May 2016	Year 1			
8	May−Jun 2016	Year 1			
9	Jun−Jul 2016	Year 1			
10	Nov−Dec 2016	Year 2	Year 1		
11	Apr−May 2017	Year 2	Year 1		
12	May−Jun 2017	Year 2	Year 1		
13	Jun 2017	Year 2	Year 1		
14	May 2018	Year 3	Year 2	Year 1	
15	Mar 2019		Year 3	Year 2	Year 1
16	May−Jun 2019		Year 3	Year 2	Year 1
17	Nov 2019			Year 3	Year 2
18	Nov−Dec 2019			Year 3	Year 2
19	Dec 2019			Year 3	Year 2
20	Jun 2020				Year 3

**Table 4:** Percentage of respondents holding a position on the left-right scale, by experience of HE and age

	Under 25		26−35		36−45		46−55		56−65		66+	
	No HE	HE	No HE	HE	No HE	HE	No HE	HE	No HE	HE	No HE	HE
0 (Left)	5.7	7.4	3	4	1.5	2.6	1.4	2.4	1.5	2.1	0.8	2
1	4.7	6.1	1.3	3.3	2.1	2.4	1	2.4	1.3	1.8	0.8	2.3
2	5.9	13.8	3.2	10.6	2.5	7.5	3.1	6.8	3.3	8	1.7	6.6
3	12.5	17	5.3	17.2	6	13.7	5	12.7	5.1	12.5	3.8	9.1
4	6.2	9.9	2.8	9.1	5	9.8	5.1	9.9	5.7	9.9	4.5	8.4
5	10.1	9.6	15.4	12.5	21.5	17.6	23.5	20.7	22.3	20.3	23.5	20.6
6	4.2	6.8	5.7	7	6.1	9.5	9.2	10.3	9.3	11.4	9.7	11.7
7	7.8	6.3	5	5	7.3	6.9	8.2	9.3	10.9	10.7	13.5	13.6
8	4.2	4	2.7	2.9	4	4.1	6.5	4.7	8.2	7.6	10.9	11.6
9	2.2	1.1	1.7	0.7	0.6	1.1	1.4	1.2	2.4	2	3.4	2.6
10 (Right)	1.8	0.9	2.3	1.1	1.7	1.5	2.5	1.9	2.4	1.6	3.9	2.8
Don’t Know	34.8	17.1	51.5	26.6	41.7	23.4	33.1	17.7	27.7	12	23.6	8.6

**Table 5:** Chi-squared test for significance on left-right identities by experience of HE. A Pearson’s X^2^ with Rao and Scott adjustment is used to compare those with and without experience of HE for each age group. Excludes ‘Don’t Know’ in the test.

Age Group	X^2^	Df	*P*-value
Under 25	35.719	10	0.0489
26–35	146.7	10	< 2.2e–16
36–45	127.27	10	3.008.2e−13
46–55	159.87	10	< 2.2e–16
56–65	161.76	10	< 2.2e–16
66+	261.78	10	< 2.2e–16

**Table 6:** Percentage of respondents holding a position on ethnocentrism (attitudes to cultural impact of immigration), by experience of HE and age

	Under 25		26–35		36–45		46–55		56–65		66+	
	No HE	HE	No HE	HE	No HE	HE	No HE	HE	No HE	HE	No HE	HE
1 (Anti)	10.1	2.9	12.9	4.2	15.5	6.1	18.1	11.3	21.4	11.3	20.2	13.3
2	9.2	2.9	7.8	4.8	9.9	4.8	11.1	7.9	10.6	9.8	12.3	8.9
3	8.2	5.7	7.9	5.3	11.2	7	11.1	8.2	12.8	9.3	12.3	10.2
4	11.1	9.4	15.7	9.8	16	12.9	17.7	13.8	14.3	13.4	17.6	15
5	11.2	13.3	14.6	17.7	13.2	17.4	14.4	16.5	14.4	15.2	14.6	17
6	12.9	19.4	9.5	22.4	11.7	20.5	9.6	17.3	10.5	16.9	9.8	17.3
7 (Pro)	24.9	41.2	12.2	29.4	10.6	25.5	8.8	21	9	20.7	6.4	15.6
Don’t Know	12.4	5.3	19.3	6.4	12	5.9	9.2	4.1	7	3.5	6.9	2.7

**Table 7:** Chi-squared test for significance on attitudes to cultural impact of immigration by experience of HE. A Pearson’s X^2 ^with Rao and Scott adjustment is used to compare those with and without HE for each age group. Excludes ‘Don’t Know’ in the test

Age Group	X^2^	Df	*P*-value
Under 25	109.9	6	5.99e−11
26−35	176.51	6	< 2.2e−16
36−45	206.18	6	< 2.2e−16
46−55	205.48	6	< 2.2e−16
56−65	224.21	6	< 2.2e−16
66+	264.4	6	< 2.2e−16

**Table 8:** Polynomial contrasts test of changes in left-right identity of individual students over time. Generalised linear model: leftRight ~ ID + Time. Where: • *leftRight* is numerical value of identity on 11-point left-right scale from 0−10, excluding those who answer ‘Don’t know’. • *ID* is unique identified used in the BES dataset. • *Time* an ordered categorical variable with the values: Year 1, Year 2, Year 3

	Estimate	Standard Error	T value	*P* value
Time Linear	–1.178e−01	4.781e−02	–2.464	0.013936
Time Quadratic	–2.135e−02	4.737e−02	–0.451	0.652349

**Table 9:** Polynomial contrasts test of changes in attitudes to cultural impact of immigration of individual students over time. Generalised linear model: immigCultural ~ ID + Time. Where: • *immigCultural* is numerical value of identity on 8-point ethnocentrism scale from 0-7, excluding those who answer ‘Don’t know’. • *ID* is unique identified used in the BES dataset. • *Time* an ordered categorical variable with the values: Year 1, Year 2, Year 3

	Estimate	Standard Error	T value	*P* value
Time Linear	2.282e−01	4.079e−02	5.594	2.90e−08
Time Quadratic	4.664e−03	4.041e−02	0.115	0.908130

**Table 10:** Comparison of changes in left-right identity and ethnocentrism of students from Year 1 to Year 3

Changes in left-right identity		Changes in ethnocentrism	
Mean movement (scale from 0=Left to 10=Right)	–0.2	Mean movement (Scale from 1=Anti to 7=Pro-immigration)	+0.4
Changes in those identifying as left, excluding ‘Don’t Know’ (% points)	+3.1	Changes in those identifying as pro-immigration, excluding ‘Don’t Know’ (% points)	+8.1
Changes in those identifying as centrist, excluding ‘Don’t Know’ (% points)	–0.1	Changes in those identifying as centrist, excluding ‘Don’t Know’ (% points)	–2.7
Changes in those identifying as right, excluding ‘Don’t Know’ (% points)	–3.0	Changes in those identifying as anti-immigration, excluding ‘Don’t Know’ (% points)	–5.4
Changes in those identifying as left, including ‘Don’t Know’ (% points)	+4.1	Changes in those identifying as pro-immigration, including ‘Don’t Know’ (% points)	+7.5
Changes in those identifying as centrist, including ‘Don’t Know’ (% points)	+1.2	Changes in those identifying as centrist, including ‘Don’t Know’ (% points)	–2.4
Changes in those identifying as right, including ‘Don’t Know’ (% points)	–1.7	Changes in those identifying as anti-immigration, including ‘Don’t Know’ (% points)	–4.9
Changes in those identifying as left, excluding ‘Don’t Know’ (% points)	–3.5	Changes in those identifying as Don’t Know (% points)	–0.2

**Table 11:** Changes in left-right identity of students from Year 1 to Year 3, compared with average differences in left-right identity of those with and without experience of HE for different age groups

Changes in left-right identity for students from Year 1 to Year 3		Average difference between those with HE and without HE for different age groups		Student change as a percentage of the overall difference
Mean movement (scale from 0=Left to 10=Right)	–0.2	Mean movement (scale from 0=Left to 10=Right)	–1.0	20.0
Changes in those identifying as left, excluding ‘Don’t Know’ (% points)	+3.1	Average difference in those identifying as left, excluding ‘Don’t Know’ (% points)	+13.8	22.5
Changes in those identifying as centrist, excluding ‘Don’t Know’ (% points)	–0.1	Average difference in those identifying as centrist, excluding ‘Don’t Know’ (% points)	–5.6	1.8
Changes in those identifying as right, excluding ‘Don’t Know’ (% points)	–3.0	Average difference in those identifying as right, excluding ‘Don’t Know’ (% points)	–8.2	36.6
Changes in those identifying as left, including ‘Don’t Know’ (% points)	+4.1	Average difference in those identifying as left, including ‘Don’t Know’ (% points)	+15.3	26.8
Changes in those identifying as centrist, including ‘Don’t Know’ (% points)	+1.2	Average difference in those identifying as centrist, including ‘Don’t Know’ (% points)	+4.2	28.6
Changes in those identifying as right, including ‘Don’t Know’ (% points)	–1.7	Average difference in those identifying as right, including ‘Don’t Know’ (% points)	–1.7	100.0
Changes in those identifying as Don’t Know	–3.5	Average difference in those identifying as Don’t Know	–17.8	19.7

**Table 12:** Changes in attitudes to cultural impact of immigration of students from Year 1 to Year 3, compared with average differences in attitudes to immigration of those with and without experience of HE for different age groups

Changes in ethnocentrism for students from Year 1 to Year 3		Average difference between those with HE and without HE for different age groups		Student change as a percentage of the overall difference
Mean movement (Scale from 1=Anti to 7=Pro-immigration)	+0.4	Mean difference (Scale from 1=Anti to 7=Pro-immigration)	+1.2	33.3
Changes in those identifying as pro-immigration, excluding ‘Don’t Know’ (% points)	+8.1	Average difference in those identifying as pro-immigration, excluding ‘Don’t Know’ (% points)	+22.6	35.9
Changes in those identifying as centrist, excluding ‘Don’t Know’ (% points)	–2.7	Average difference in those identifying as centrist, excluding ‘Don’t Know’ (% points)	–4.4	61.4
Changes in those identifying as anti-immigration, excluding ‘Don’t Know’ (% points)	–5.4	Average difference in those identifying as anti-immigration, excluding ‘Don’t Know’ (% points)	–18.2	29.7
Changes in those identifying as pro-immigration, including ‘Don’t Know’ (% points)	+7.5	Average difference in those identifying as pro-immigration, including ‘Don’t Know’ (% points)	+24.3	30.9
Changes in those identifying as centrist, including ‘Don’t Know’ (% points)	–2.4	Average difference in those identifying as centrist, including ‘Don’t Know’ (% points)	–3.0	80.0
Changes in those identifying as anti-immigration, including ‘Don’t Know’ (% points)	–4.9	Average difference in those identifying as anti-immigration, including ‘Don’t Know’ (% points)	–14.8	33.1
Changes in those identifying as Don’t Know (% points)	–0.2	Average difference in those identifying as Don’t Know (% points)	–6.5	3.1

## Footnotes

1 An alternative grouping would be 1–2 as ‘Anti-immigration’, 3–5 as ‘Centre’ and 6–7 as ‘Pro-immigration’. The findings and patterns are similar across both coding approaches. On balance, this paper decided to restrict the ‘Centre’ to 4. This more restricted ‘Centre’ category allows a comparison of those expressing any pro- (5–7) or anti-immigration (1–3) perspectives, rather than limiting this to those with *strong* pro- (6–7) and anti-immigration (1–2) views.

2 This paper focuses on the attitudes to the cultural impacts of immigration, although a similar pattern is seen if a second question from the BES, on perceptions of the economic impacts of immigration, is considered.

3 This analysis excluded the 173 students (30.1% of the 574 sample) who said ‘Don’t Know’ in Year 1 and/or Year 3. This data was excluded to allow the quantification of movement on the scale, shown in Fig. 4. Of the students who reported ‘Don’t Know’ in Year 1, 66.9% also reported ‘Don’t Know’ in Year 3; this stability implies the exclusion has little impact on the overall conclusions of Fig. 4.
